# Relationships between parental sleep quality, fatigue, cognitions about infant sleep, and parental depression pre and post-intervention for infant behavioral sleep problems

**DOI:** 10.1186/s12884-017-1284-x

**Published:** 2017-04-04

**Authors:** Wendy A. Hall, Melissa Moynihan, Radhika Bhagat, Joanne Wooldridge

**Affiliations:** 1 University of British Columbia School of Nursing, T. 201, 2211 Wesbrook Mall, Vancouver, BC V6T 2B5 Canada; 2 School of Nursing, University of British Columbia, Vancouver, BC Canada; 3 South Community Health Centre, Vancouver Coastal Health, Vancouver, BC Canada; 4 Maternal Child Program, Vancouver Coastal Health, Vancouver, BC Canada

**Keywords:** Depression, Sleep cognitions, Infant, Sleep problems, Fatigue

## Abstract

**Background:**

Maternal and paternal depression has been associated with infants’ behavioral sleep problems. Behavioral sleep interventions, which alter parental cognitions about infant sleep, have improved infant sleep problems. This study reports relationships between parental depression, fatigue, sleep quality, and cognitions about infant sleep pre and post-intervention for a behavioral sleep problem.

**Methods:**

This secondary analysis of data from Canadian parents (*n* = 455), with healthy infants aged 6-to-8-months exposed to a behavioral sleep intervention, examined baseline data and follow-up data from 18 or 24 weeks post intervention (group teaching or printed material) exposure. Parents reported on sleep quality, fatigue, depression, and cognitions about infant sleep. Data were analyzed using Pearson’s r and stepwise regression analysis.

**Results:**

Parents’ fatigue, sleep quality, sleep cognitions, and depression scores were correlated at baseline and follow-up. At baseline, sleep quality (*b* = .52, 95% *CI* .19–.85), fatigue (*b* = .48, 95% *CI* .33–.63), doubt about managing infant sleep (*b* = .44, 95% *CI* .19–.69), and anger about infant sleep (*b* = .69, 95% *CI* .44–.94) were associated with mothers’ depression. At baseline, fathers’ depression related to sleep quality (*b* = .42, 95% *CI* .01–.83), fatigue (*b* = .47, 95% *CI* .32–.63), and doubt about managing infant sleep (*b* = .50, 95% *CI* .24–.76). At follow-up, mothers’ depression was associated with sleep quality (*b* = .76, 95% *CI* .41–1.12), fatigue (*b* = .25, 95% *CI* .14–.37), doubt about managing infant sleep (*b* = .44, 95% *CI* .16–.73), sleep anger (*b* = .31, 95% *CI* .02–.59), and setting sleep limits (*b* = −.22, 95% *CI* -.41-[−.03]). At follow-up, fathers’ depression related to sleep quality (*b* = .84, 95% *CI* .46–1.22), fatigue (*b* = .31, 95% *CI* .17–.45), sleep doubt (*b* = .34, 95% *CI* .05–.62), and setting sleep limits (*b* = .25, 95% CI .01–.49).

**Conclusions:**

Mothers’ and fathers’ cognitions about infant sleep demonstrate complex relationships with their depression scores. While mothers’ setting sleep limit scores are associated with decreased depression scores, fathers’ setting limits scores are associated with increased depression scores. Parental doubts about managing infant sleep and difficulties with setting sleep limits require attention in interventions.

## Background

Parenting is a very complex area, particularly in the context of parent-identified infant behavioral sleep problems. Twenty to 30% of young children have sleep disturbances [1]. Sleep problems can persist for about 30% of children, particularly infants [2]. Behavioral sleep problems are evident when infants’ sleep time is shorter than age-appropriate norms and infants show evidence of sleep loss [3]. The presence of a behavioral sleep problem also depends on whether parents define infants’ sleep as a problem [1]; parents commonly view frequent infant night waking with crying beyond 6 months of age as a problem [4].

Maternal stress, depressive symptoms, and poorer self-reported health [5], and poor paternal general health [6] and depressive symptoms have been associated with infants’ behavioral sleep problems [7, 8]. Moreover, in mothers without a past history of depression, infant sleep problems have had a greater impact on mothers’ severe psychological distress [6]. Fathers attending a service for early parenting difficulties reported higher levels of distress when children had more severe sleep problems [9]. Children’s sleep problems persisting from infancy or recurring in preschoolers have been associated with higher maternal depression scores [10].

For parents, fatigue, sleep quality, and psychological distress (including depression) are associated. Mothers and fathers have reported high levels of fatigue with parental sleep quality contributing to fatigue in the first 6 months post birth [11]. Poorer sleep quality has also been associated with higher parental fatigue levels in families with infants, toddlers, and preschoolers [9, 12]. Fatigue in the postnatal period accounted for 59% of the variance in mothers’ depression scores [13]. Maternal fatigue at 12 months post-birth predicted depression scores at 18 months post-birth [14]. In early parenthood, poor sleep quality contributed to variance in fathers’ anxiety and stress but not depression scores [15]; because parental sleep quality, fatigue, and parental distress, including depression, are linked, it is important to examine contributions of parents’ sleep quality and fatigue to depression. This is particularly important for fathers who have received minimal research attention.

Parental cognitions (expectations and attitudes) about infant sleep, in particular doubts about managing infant sleep, difficulty with setting sleep limits, and anger at infants’ demands around sleep, have consistently been associated with infant behavioral sleep problems [16–19]. When maternal cognitions about sleep and depression for mothers of 31 children with behavioral sleep problems and 170 control mothers were compared, mothers of children with sleep problems reported more doubts about managing infant sleep, anger at infants’ demands around sleep, and depressive symptoms than controls [20]. Mothers with higher levels of depressive symptoms have reported more infant night waking and more maternal worries (cognitions) about adequately responding to their infants' needs at night [21].

Because parental cognitions drive parents’ behaviors around infant sleep, modifying their cognitions is a major element in behavioral interventions to improve infants’ sleep [1]. Regardless of whether maternal depression precedes infant sleep problems or infant sleep problems precede depression, an area of great debate in the literature, [22] interventions to resolve infant behavioral sleep problems have improved infant night waking [4, 23, 24] and maternal and paternal depression scores post intervention [8, 25–27]. Moreover, mothers of infants exposed to a preventative sleep intervention reported lower depression scores and fewer doubts (cognition) about managing their infants’ sleep problems than a control group [28].

Parental depression has implications for many parenting interactions with infants. Caregiving by mothers with depression can involve more intrusive or withdrawn interactions, less preventative health care visits (immunization), and less satisfaction with breastfeeding [29]. Maternal postnatal depression has been associated with increased paternal depression and parenting stress, and less optimal father-infant interactions [30]. Paternal depressive symptoms have been linked with children’s internalizing and externalizing behaviors in early childhood [31].

Relationships between parental depression scores, fatigue, sleep quality, and infant sleep cognitions require examination. Using baseline data (prior to group assignment and intervention exposure) and follow-up data (post intervention exposure) from mothers and fathers of healthy 6–8 month-old-infants with behavioral sleep problems, this secondary analysis aimed to: 1) identify proportions of mothers and fathers reporting high and clinically significant depressive symptoms at baseline and follow-up; 2) identify mothers’ and fathers’ means and standard deviations for depression, sleep quality, fatigue, and sleep cognitions (baseline, follow-up and change scores); 3) examine relationships between mothers’ and fathers’ depression, sleep quality, fatigue, and sleep cognitions scores at baseline and follow-up; 4) explore the variance in mothers’ and fathers’ depression scores explained by sleep quality, fatigue, and sleep cognitions scores at baseline and follow-up; and 5) explore the variance in mothers’ and fathers’ change in depression scores explained by changes in sleep quality, fatigue, and sleep cognition scores from baseline to follow-up.

## Methods

The study, from which these data are drawn for secondary analysis, was a randomized controlled trial to evaluate a cognitive-behavioural sleep intervention for healthy 6-to-8-months-old infants based on improvement in parental perceptions of infant sleep problems and reduced infant night waking at 6 weeks post-intervention [8]. The study sample was recruited between September 2009 and March 2011. Parents, who reported a diagnosis of depression or receiving treatment for depression (anti-depressant medications or cognitive therapy), had diagnosed sleep problems, or worked permanent night shifts, were excluded. An infant sleep problem was defined as an infant waking two or more times per night and/or wakes lasting more than 20 min, occurring at least four nights per week for a minimum of 3 weeks [32]. Two hundred and thirty-five families (including 8 single-parent families) provided baseline data and 188 families provided follow-up data. This secondary analysis reports combined control and intervention group data from baseline (before randomization) and outcomes assessed at 18–24 weeks post-intervention exposure (follow-up).

A brief summary of the trial follows; details about the trial have been published elsewhere [8]. The primary outcome measure was significant infant sleep disturbance: parent report of a severe sleep problem or actigraphic wakes of greater than two per night averaged over five nights. Secondary outcomes were improvements in parents’ perceptions of their mood, fatigue, and sleep quality, and the quality of their cognitions about infant sleep. Following randomization into intervention and control groups, both groups received a 2-h teaching session and 2 weeks of bi-weekly telephone support calls. The intervention group received information about managing infant sleep problems and the control group received information about managing infant safety risks. Trial outcomes were assessed at 6 weeks post-teaching session. Following that assessment, the sleep group received a pamphlet summarizing the safety information and the safety group received a pamphlet reproducing the information taught in the sleep intervention training session.

At baseline and follow-up, parents completed questionnaires which included demographic questions, and measures of parental sleep quality, fatigue, depression, and cognitions about infant sleep. Parents provided follow-up data at 24 weeks post-intervention exposure (sleep and safety training); however, because the control group was exposed to the sleep intervention (printed material) at 6 weeks post-intervention their data collection occurred at 18 weeks post sleep intervention.

In the study database, parental designation was by primary and secondary caregiver because one caregiver took the lead for the intervention. In only one two-parent family, the primary caregiver was male and all single parents were females; therefore, we refer to mothers (primary caregivers) and fathers (secondary caregivers) in this paper. One family self-identified as a lesbian couple. At follow-up, 188 mothers and 171 fathers responded with questionnaire data. Because this is a secondary analysis of data obtained for a trial, power calculations were not undertaken for examining relationships among psychological variables.

### Measures

At baseline, data were collected on parents’ age, marital/partner status, years of formal education, number of children, work status and hours of work, family income, rating of infant sleep problem severity, and ethnicity. Data were also collected on infant gender, birth order, age, breast feeding status, and co-sleeping. At follow-up, only data about parents’ age, length of relationship, highest level of education, and ethnicity and infant gender were not collected again.

The Centre for Epidemiologic Studies Depression Scale (CESD) was used to measure depressive symptoms [33]. Higher scores indicated higher levels of symptoms, with a score of ≥16 being used as the cut-off point for high depressive symptoms and ≥22 being used to indicate clinically significant depression. The CESD has demonstrated reliability and validity when used with childbearing populations [34]. In a quasi-experimental intervention study for infants’ behavioral sleep problems, the measure indicated a significant improvement in mood following the intervention (*p* = .001) [25]. In this study, Cronbach’s alpha ranged from 0.89 to 0.90. The use of the term depression in this paper is not intended to indicate a diagnosis of clinical depression.

The Pittsburgh Sleep Quality Index (PSQI) was used to assess parents’ sleep quality [35]. Higher scores indicate worse sleep quality. The PSQI has demonstrated relationships between lifestyle regularity and sleep quality in healthy subjects [36]. In a quasi-experimental study of an intervention for parents with infants with behavioral sleep problems, sleep quality improved significantly post-intervention (*p* < .001) [25]. In this study, Cronbach’s alpha ranged from 0.53 to 0.69.

The Multidimensional Assessment of Fatigue Scale (MAF) Global Fatigue Index (GFI) was used to assess fatigue [37]. Higher scores equal more fatigue. Reliability and validity of the measure was supported with two large cohorts of postnatal women [38]. In a quasi-experimental study of an intervention to manage an infant sleep problem, MAF scores demonstrated significant reductions in parental fatigue post-intervention (*p* < .001) [25]. In this study, Cronbach’s alpha ranged from 0.94 to 0.95.

The Maternal Cognitions about Infant Sleep Questionnaire (MCISQ) was used to measure parents’ thoughts about managing infant sleep [16]. The questionnaire comprises 20 items with five subscales including: limit setting around infant sleep, anger about sleep, doubt about managing infant sleep, the necessity of feeding infants at night, and infant sleep safety. Higher scores indicate more difficulty with managing sleep. The MCISQ has predicted settling strategies and infant sleep problems [17, 39] and differentiated infants with poor and good sleep quality using parental difficulty with limit setting [18]. In a small quasi-experimental study using the MCISQ, parents’ cognitions improved after a sleep intervention for infant behavioral sleep problems including: setting sleep limits (*p* < .001, *d* = 1), anger about sleep problems (*p* < .001, *d* = 0.7), doubt about handling sleep problems (*p* < .001, *d* = 0.7), and necessity for night feeding (*p* < .001, *d* = 1) [25]. In this study, Cronbach’s alpha for the subscales ranged from 0.5-0.75.

Parental perceptions about infant sleep were measured using the Child Sleep Question from the Longitudinal Study of Australian Children (LSAC). The measure has been used in a number of studies to indicate the extent to which parents perceived their child to have a sleep problem (no, mild, moderate, or severe) [40–43].

### Statistical analysis

For baseline and follow-up, we calculated univariate descriptive statistics (means, standard deviations) and normality statistics for continuous, demographic, and subscale data, and percentages for categorical data. We examined changes in proportions of parents with depressive symptomatology and clinically depressive symptomatology based on cut-off scores on the CESD. We calculated change scores (follow-up - baseline) for all of the variables and used Pearson’s r to examine correlations among continuous variables.

To examine mothers’ and fathers’ predictors of depression, we undertook six forward stepwise multiple regression analyses, two at baseline, two at follow-up and two using change scores to determine the best combination of sleep quality, fatigue, and cognitions about infant sleep for predicting depression. To control for intervention/control group allocation, group assignment was entered as an independent variable in all of the models; we used change scores for one model. We entered all eight of the independent variables (group, fatigue, sleep quality, sleep doubts, sleep limits, sleep anger, sleep safety, and sleep and feeding) simultaneously. When the backward method was used the results were the same. The assumptions were met for performing multiple regression analysis. No two independent variables included in the models had an association of *r* ≥ 0.60, minimizing potential for multi-collinearity. Checks of assumptions yielded no indicators of multicollinearity. The outcome variables at follow-up were slightly heteroscedastic. We used IBM SPSS Statistics for Windows, version 24 (IBM Corp, Armonk, N.Y., USA) and considered p-values <0.05 as statistically significant. Correlation analyses were two-sided. We compared observed proportions of parents reporting none-mild sleep problems using a Fisher’s exact test with adjustment for baseline severity using the Mantel-Haenszel test, including corresponding 95% confidence intervals for differences in proportions. We used ANOVA to examine change of scores between baseline and follow-up for primary and secondary caregivers.

## Results

Demographic data at baseline and follow-up demonstrate similar characteristics (See Tables 1 and 2). By follow-up, most mothers had returned to paid employment. Given the infants’ ages were between 11 and 16 months at follow-up the proportion of infants still breastfeeding was very high (55.2%). At follow-up, most mothers identified infant sleep as not a problem or a mild problem (72.3%) compared with baseline where the majority identified a moderate to severe problem (81%). A minority of parents (26.2%) were co-sleeping at follow-up. We used Chi-Square to compare parents by missing data on their CESD scores at follow-up based on their demographic characteristics at baseline; there were no significant differences on family income, education, parents’ age, partner status, sleep problem rating, or cultural identity.

**Table 1:** Mother and father baseline demographic variables

	Mothers *n* = 232	Fathers *n* = 223
Age in years, [M (SD)], range	34.4 (4.5), (22, 54)	36.5 (5.9), (22, 60)
Years of education, [M (SD)], range	17.5 (2.7), (10, 30)	17.1 (2.9), (4, 27)
Highest level of education, *n* (%)		
Some high school	0 (0)	1 (0.4)
High school completed	4 (1.7)	8 (3.6)
Some college	13 (5.6)	24 (10.8)
College completed	25 (10.8)	28 (12.6)
University courses	10 (4.3)	15 (6.7)
University degree	107 (46.1)	85 (38.1)
Post-graduate degree	73 (31.5)	62 (27.8)
Cultural identity^a^, *n* (%)		
American	2 (0.9)	7 (3.2)
Asian	27 (11.6)	14 (6.3)
Canadian	121 (52.2)	113 (50.9)
Chinese	19 (8.2)	23 (10.4)
European	24 (10.3)	32 (14.4)
Central or South American	5 (2.2)	3 (1.4)
Other	34 (14.6)	30 (13.5)

^a^Fathers *n* = 222

**Table 2:** Parent, infant, and family variables at baseline and follow-up

	Baseline	Follow-up
Mothers	*n = 232*	*n = 188*
Paid employment, *n* (%)	40 (17.2)	122 (64.9)
Sleep problem rating, *n* (%)		
Not a problem	1 (0.4)	72 (38.3)
Mild	43 (18.5)	64 (34.0)
Moderate	148 (63.8)	48 (25.5)
Severe	40 (17.2)	4 (2.1)
Fathers	*n = 223*	*n = 171*
Paid employment, *n* (%)	205 (91.9)	159 (93.0)
Sleep problem rating, *n* (%)		
Not a problem	8 (3.6)	63 (36.8)
Mild	42 (18.8)	58 (33.9)
Moderate	139 (62.3)	48 (28.1)
Severe	34 (15.2)	2 (1.2)
Infants^a^	*n = 455*	*n = 359*
Age in months, [M (SD)], range	6.7 (0.9), (5, 10)	12.9 (1.1), (11, 16)
Breastfed, *n* (%)	410 (90.1)	198 (55.2)
Co-sleeping, *n* (%)	275 (60.4)	94 (26.2)
Family^a^	*n = 440*	*n = 350*
Income, *n* (%)		
$10,000 – 29,999	20 (4.5)	16 (4.6)
$30,000 – 59,999	64 (14.5)	47 (13.4)
$60,000 – 89,999	81 (18.4)	72 (20.6)
$90,000 – 109,999	91 (20.7)	64 (18.3)
More than $110,000	184 (41.8)	151 (43.1)

^a^Responses from both mothers and fathers

At baseline, mothers’ mean depression scores exceeded the cut-off for the CESD for high depressive symptoms but the fathers’ mean scores did not (See Table 3). The mean change scores indicated that all of the variables (higher scores = more difficulties) changed in the desired direction. Mothers’ and fathers’ means on the CESD were equivalent at follow-up.

**Table 3:** Means (SDs), ranges and change scores for key variables at baseline and follow-up

	Mothers		Fathers	
	M (SD)	Range	M (SD)	Range
Baseline	*n = 231*		*n = 217*	
CESD	16.4 (9.5)	(0, 39)	13.0 (9.7)	(0, 48)
PSQI	9.7 (3.3)	(2, 19)	6.5 (2.9)	(1, 16)
MAF	30.4 (7.2)	(8.4, 43.4)	23.2 (8.7)	(5, 41.2)
Setting sleep limits	16.5 (4.5)	(5, 25)	13.1 (4.9)	(0, 25)
Sleep doubts	8.0 (3.9)	(0, 20)	6.1 (4.3)	(0, 18)
Sleep anger	7.0 (3.9)	(0, 18)	6.9 (3.5)	(1, 25)
Sleep feeding	8.1 (3.5)	(0, 15)	5.9 (3.5)	(0, 15)
Sleep safety	3.0 (2.4)	(0, 10)	2.6 (2.4)	(0, 10)
Follow-up	*n = 185*		*n = 170*	
CESD	9.2 (7.5)	(0, 34)	9.2 (8.0)	(0, 45)
PSQI	5.8 (2.9)	(0, 15)	5.2 (3.0)	(0, 15)
MAF	18.2 (9.0)	(5.0, 38.5)	17.8 (8.7)	(5.0, 42.7)
Setting sleep limits	9.9 (4.9)	(1, 25)	8.8 (4.3)	(0, 25)
Sleep doubts	4.1 (3.5)	(0, 16)	3.5 (3.6)	(0, 15)
Sleep anger	5.0 (3.3)	(0, 19)	4.9 (3.1)	(0, 16)
Sleep feeding	3.0 (3.3)	(0, 14)	2.7 (3.1)	(0, 14)
Sleep safety	1.8 (2.0)	(0, 8)	1.5 (2.0)	(0, 10)
Change scores	*n = 185*		*n = 169*	
CESD	−6.4 (9.1)	(−33, 19)	−3.1 (8.6)	(−42, 19)
PSQI	−4.0 (3.5)	(−13, 5)	−1.4 (2.9)	(−9, 8)
MAF	−11.8 (10.2)	(−34.4, 21.9)	−5.3 (10.0)	(−27.2, 17.4)
Setting sleep limits	−6.4 (4.9)	(−18, 5)	−4.1 (4.7)	(−19, 7)
Sleep doubts	−3.7 (4.1)	(−15, 5)	−2.4 (3.9)	(−12, 15)
Sleep anger	−2.2 (3.6)	(−14, 8)	−2.1 (3.5)	(−16, 9)
Sleep feeding	−4.9 (4.0)	(−14, 6)	−3.1 (3.7)	(−11, 9)
Sleep safety	−1.1 (2.2)	(−8, 4)	−1.0 (2.2)	(−7, 7)

*Abbreviations: CESD* depression, *PSQI* sleep quality, *MAF* fatigue

*Note:* Means and SDs values from descriptive statistics of regression models; range values from descriptive statistics; change scores calculated by subtracting baseline from follow-up values

At baseline, 47.8% (*n* = 111) of mothers and 34.5% (*n* = 76) of fathers reported CESD scores ≥16. At follow-up, only 18.1% (*n* = 34) of mothers and 14.6% (*n* = 25) of fathers reported CESD scores of ≥16. At baseline, 29.7% (*n* = 69) of mothers and 18.6% (*n* = 41) of fathers reported CESD scores ≥22, while, at follow-up, only 8.5% (*n* = 16) of mothers and 7.6% (*n* = 13) of fathers reported CESD scores of ≥22.

At baseline (*n* = 455, mothers and fathers) and follow-up (*n* = 359, mothers and fathers), parental fatigue, sleep quality, doubts about managing infant sleep, difficulties with sleep and feeding, anger about infant sleep, sleep safety, and difficulties setting sleep limits for infants were significantly positively correlated with depression scores. The cognition scores were also significantly positively correlated with parental sleep quality and fatigue. Sleep quality, fatigue and depression were moderately correlated (See Table 4).

**Table 4:** Correlations among variables for all participants at baseline and follow-up

	Correlation coefficients baseline								Correlation coefficients follow-up							
	1	2	3	4	5	6	7	8	1	2	3	4	5	6	7	8
1. SD	1								1							
2. SF	.42	1							.47	1						
3. SL	.48	.43	1						.44	.45	1					
4. Ang	.34	.20	.09*	1					.39	.35	.26	1				
5. SS	.41	.26	.23	.14	1				.47	.35	.29	.33	1			
6. PSQI	.28	.27	.26	.13	.16	1			.21	.24	.20	.24	.24	1		
7. MAF	.38	.29	.29	.19	.22	.62	1		.29	.33	.27	.36	.27	.58	1	
8. CESD	.43	.23	.23	.34	.27	.46	.58	1	.35	.31	.22	.33	.30	.53	.58	1

Correlations *p* < 0.01, 2-tailed; * indicates *p* > 0.05

*Abbreviations:* 1) *SD* Sleep Doubt; 2) *SF* Sleep & Feeding; 3) *SL* Setting Sleep Limits; 4) *Ang* Sleep Anger 5) *SS* Sleep Safety; 6) *PSQI* sleep quality; 7) *MAF* fatigue; 8) *CESD* depression

At baseline, mothers’ depression was associated with sleep quality (*b* = .52), fatigue (*b* = .48), doubt about managing infant sleep (*b* = .44), and anger about infant sleep (*b* = .69). The model accounted for 47% of the variance (see Table 5). At baseline, fathers’ depression related to sleep quality (*b* = .42), fatigue (*b* = .47), and doubt about managing infant sleep (*b* = .50). The model accounted for 39% of the variance (See Table 6).

**Table 5:** Stepwise multiple regression analysis explaining mothers’ depression from sleep quality, fatigue, and sleep cognitions

	Unstd B (SE)	t	Sig.	95% CI for B	Cumulative R^2^
Baseline (*N* = 231)					
Constant	−11.63 (2.10)	−5.53	<.001	[−15.77, −7.48]	
MAF	.48 (.08)	6.28	<.001	[.33, .63]	.31
Sleep doubt	.44 (.13)	3.49	.001	[.19, .69]	.42
PSQI	.52 (.17)	3.12	.002	[.19, .85]	.46
Sleep anger	.69 (.13)	5.52	<.001	[.44, .94]	.48
Follow-up (*N* = 185)					
Constant	-.92 (1.26)	-.73	.467	[−3.41, 1.57]	
MAF	.25 (.06)	4.27	<.001	[.14, .37]	.28
PSQI	.76 (.18)	4.26	<.001	[.41, 1.12]	.34
Sleep doubt	.44 (.14)	3.08	.002	[.16, .73]	.38
Setting sleep limits	-.22 (.10)	−2.26	.025	[−.41, −.03]	.39
Sleep anger	.31 (.15)	2.10	.037	[.02, .59]	.41

*Abbreviations: CI* confidence interval, *PSQI* sleep quality, *MAF* fatigue

Baseline: *R* ^2^ = .48, adjusted *R* ^2^ = .47, *F* (4, 226) = 51.9, *p* < .001

Follow-up: *R* ^2^ = .41, adjusted *R* ^2^ = .39, *F* (5, 179) = 24.6, *p* < .001

**Table 6:** Stepwise multiple regression analysis explaining fathers’ depression from sleep quality, fatigue, and sleep cognitions

	Unstd B (SE)	t	Sig.	95% CI for B	Cumulative R^2^
Baseline (*N* = 217)					
Constant	−3.79 (1.57)	−2.42	.017	[−6.88, −.70]	
MAF	.47 (.08)	6.20	<.001	[.32, .63]	.34
Sleep doubt	.50 (.13)	3.77	<.001	[.24, .76]	.38
PSQI	.42 (.21)	2.00	.046	[.01, .83]	.39
Follow-up (*N* = 170)					
Constant	−4.00 (1.25)	−3.19	.002	[−6.47, −.1.53]	
MAF	.31 (.07)	4.37	<.001	[.17, .45]	.39
PSQI	.84 (.19)	4.39	<.001	[.46, 1.22]	.44
Sleep doubt	.34 (.15)	2.29	.023	[.05, .62]	.48
Setting sleep limits	.25 (.12)	2.06	.041	[.01, .49]	.49

*Abbreviations: CI* confidence interval, *PSQI* sleep quality, *MAF* fatigue

Baseline: *R* ^2^ = .39, adjusted *R* ^2^ = .39, *F* (3, 213) = 46.1, *p* < .001

Follow-up: *R* ^2^ = .49, adjusted *R* ^2^ = .48, *F* (4, 165) = 39.9, *p* < .001

At follow-up, mothers’ depression was associated with sleep quality (*b* = .76), fatigue (*b* = .25), doubt about managing infant sleep (*b* = .44), sleep anger (*b* = .31), and setting sleep limits (*b* = −.22). The model accounted for 39% of the variance (see Table 5). At follow-up, fathers’ depression was linked to sleep quality (*b* = .84), fatigue (*b* = .31), sleep doubt (*b* = .34), and setting sleep limits (*b* = .25). The model accounted for 48% of the variance (see Table 6).

When change scores were examined change in fatigue (*b* = .25), sleep anger (*b* = .59), sleep quality (*b* = .53), and sleep doubt (*b* = .38) was associated with change in maternal depression scores, with the model accounting for 33% of the variance. Fathers’ change in depression scores related to changes in fatigue (*b* = .36), sleep safety (*b* = .89), and sleep anger scores (*b* = .37), with the model accounting for 35% of the variance (See Table 7).

**Table 7:** Stepwise multiple regression analysis explaining caregivers' change in depression from changes in sleep quality, fatigue, and sleep cognitions

	Unstd B (SE)	t	Sig.	95% CI for B	Cumulative R^2^
Mothers (*N* = 185)					
Constant	1.26 (.98)	1.28	.201	[−.68, 3.21]	
MAF change	.25 (.06)	3.83	<.001	[.12, .37]	.22
Sleep anger change	.59 (.16)	3.74	<.001	[.28, .90]	.28
PSQI change	.53 (.18)	2.90	.004	[.17, .89]	.32
Sleep doubt change	.38 (.14)	2.74	.007	[.11, .65]	.34
Fathers (*N* = 169)					
Constant	.43 (.67)	.64	.521	[−.89, 1.74]	
MAF change	.36 (.06)	5.97	<.001	[.24, .47]	.29
Sleep safety change	.89 (.26)	3.45	.001	[.38, 1.40]	.34
Sleep anger change	.37 (.16)	2.28	.024	[.05, .70]	.36

*Abbreviations: CI* confidence interval, *PSQI* sleep quality, *MAF* fatigue

Mothers: *R* ^2^ = .34, adjusted *R* ^2^ = .33, *F* (4, 180) = 23.5, *p* < .001

Fathers: *R* ^2^ = .36, adjusted *R* ^2^ = .35, *F* (3, 165) = 31.4, *p* < .001

## Discussion

Our results demonstrated reductions in parents’ levels of high and clinically significant depression scores from baseline to follow-up. All of the change scores reported indicated reductions in fatigue, poor sleep quality, depression, and problematic parental cognitions about infant sleep from baseline to follow-up. The variables all demonstrated small to moderate statistically significant correlations. At baseline and follow-up, maternal depression was associated with fatigue, sleep quality, doubt about managing infant sleep, and sleep anger. At follow-up, setting sleep limits had a statistically significant negative relationship with maternal depression. At baseline and follow-up, paternal depression was related to fatigue, sleep quality, and doubt about managing infant sleep. At follow-up only, paternal depression was associated with setting sleep limits. To control for baseline, we also examined variables associated with mothers’ and fathers’ change in depression scores from baseline to follow-up. Mothers’ changes in depression followed a similar pattern to variables associated with depression scores. Changes in maternal fatigue, sleep quality, sleep anger and doubt about managing infant sleep related to change in maternal depression. For fathers, the pattern was quite different because changes in fatigue, sleep anger, and infant safety during sleep were associated with changes in paternal depression.

Our findings demonstrated moderate correlations among depression scores and fatigue and sleep quality scores in a sample of men and women who had not been diagnosed with or treated for depression prior to study recruitment. Moreover, sleep quality and fatigue were not only making significant contributions to depression scores at baseline but also at 18 to 24 weeks follow-up, when our infants’ mean age was 12.9 months. Change in depression scores was associated with fatigue change for mothers and fathers, although change in sleep quality was only significant for mothers. Our findings emphasize the importance of Giallo and colleagues’ work demonstrating that maternal fatigue at 12 months post-birth predicted depression scores at 18 months post-birth [14]. In other studies, infants’ sleep problems at 4 and 6 months have been associated with increased paternal depression and poor personal sleep quantity and quality [7] suggesting fathers’ mental health is also suffering. High levels of parental fatigue have also been associated with lower parenting competence, greater stress, more irritability in parent–child interactions, and poorer sleep quality [12].

Parental cognitions have also been associated with infant behavioral sleep problems [16–19] but effects of parental cognitions on depression, in combination with parental fatigue and sleep quality, are rarely studied. Our results indicated that parental cognitions consistently contributed to variance associated with maternal and paternal depression when sleep quality and fatigue were also considered, in particular, parents’ doubts about managing infant sleep. Setting sleep limits contributed to variance in depression scores for mothers and fathers only at follow-up and for mothers, the relationship was negative (less difficulty setting sleep limits associated with lower depression scores). In another study, when mothers identified children as having behavioral sleep problems, their cognitions about infant sleep were significantly positively correlated with their depression scores; doubts about infant sleep, difficulties with limit setting, and anger about infant sleep, and scores on the depression measure were significantly higher in the infant behavioral sleep problem group than in the feeding problem or control groups [20].

At baseline, but not at follow-up, mothers’ anger about infant sleep problems was contributing more variance to their depression scores than sleep quality. Fathers’ anger did not make a statistically significant contribution to depression variance at baseline or at follow-up but their change scores for sleep anger and fears about infant safety contributed to their depression change score. Parents may be fearful of losing control and harming their infants when experiencing anger about infant sleep problems. Fathers have reported anger about infants’ sleep problems at 4 and 6 months of age [7].

In our study, the patterns in relationships between cognitions and parental depression indicated that parents’ doubts about managing infant sleep persisted in contributing to depression score variance and fathers’ difficulties with setting sleep limits had a positive relationship with depression. Morrell linked infant night waking with problematic parental cognitions about limit setting, doubt, and anger to over-intrusive and rejecting parental interactions [16]. Difficulties with setting limits can persist because mothers’ difficulty setting limits for 12-months-old infants has predicted objectively measured night waking problems when children are 4 years old [44]. When Teti and Crosby tested models correlating maternal depressive symptoms, ‘dysfunctional cognitions (worries about infant needs and maternal helplessness/loss of control)’, and infant night waking, for cohorts between 5 weeks and 25.3 months, they suggested that maternal depressive symptoms, nighttime presence, and worries about infant night needs were driving infant sleep problems [21]. They also acknowledged; however, that chronic infant night waking and distress could elicit maternal interventions that increase maternal distress. Rather than endless academic speculation about whether parental depression is driving infant sleep problems or infant sleep problems are driving parental depression it is important to offer parents interventions to manage infant sleep problems.

In our study, there was a marked improvement in parents’ depression from levels at baseline to 18–24 week follow-up; there was a reduction in proportions of mothers and fathers scoring above cut-offs on the CESD for depressive symptomatology by between 11 and 30%. The literature has demonstrated that, not only have infant behavioral sleep problems been consistently associated with maternal depression scores [5, 8, 26, 27] and, under the rare circumstances where fathers are assessed, with paternal depression scores [7, 8, 25], but also that interventions for infant behavioral sleep problems have improved infant night waking [4, 8, 23] and maternal and paternal depression scores [8, 25–27]. A recent trial by Gradisar and colleagues reported that graduated extinction (also called controlled comforting) improved infant sleep and decreased infant cortisol levels and maternal stress compared with controls (sleep education) [24].

### Limitations

This secondary analysis of the trial data relies on a sample of convenience. Although we did not detect any demographic differences on missing CESD scores it is possible that retention bias could have been operating. Requiring commitment by both parents to the study reduced the likelihood of parents experiencing marital or parenting conflict participating in the study. We excluded parents with diagnosed depression or receiving treatment for depression because pre-existing chronic depression would be unlikely to be sensitive to changes in infants’ night waking but excluding those parents limits the generalizability of our findings. We combined the control group and intervention group study participants because, at follow-up, there were no significant differences between groups on depression, fatigue, or sleep scores, combining the groups increased the study power, and we wanted to examine relationships between parental cognitions and depression following interventions for infants’ behavioral sleep problems.

It is a limitation that parents were exposed to different forms of the intervention (face-to-face as opposed to written materials) at different time points. The control group received intervention materials 6 weeks later than the intervention group, which could influence the utility of the intervention. Other researchers have reported that comparisons of face-to-face treatment and exposure to written materials revealed no differences in efficacy post-intervention [45, 46]. When we compared intervention and control groups at follow-up, there were no significant differences on any of the measures except for parents’ perceptions of the severity of the infant sleep problem of none-mild (96.3% intervention versus 86% control, *p* < .001, 95% *CI* 2.8%–17.8%) and a significant improvement in intervention group fathers’ sleep doubt (*F*(1,171) = 5.6, *p* = 0.02) and comfort with sleep and feeding (*F*(1,171) = 8.8, *p* = 0.004) compared with the control group. It may be possible that fewer fathers in the control group were exposed to the sleep intervention through reading the written material.

### Strengths

We used the comparison of baseline and follow-up data to explore changes in parents’ psychological variables for a large sample of parents with infants in a narrow age range (i.e., developmentally similar). We used tools with evidence for reliability and validity for parents’ psychological measures. Our inclusion of fathers is an important step forward in understanding parents’ patterns of cognitions.

## Conclusion

Our study demonstrates complex associations between mothers’ and fathers’ depression scores, sleep quality, fatigue, and sleep cognitions. Findings suggest it is important to provide parents with ongoing support to attend to fathers’ comfort with setting limits about infant sleep and mothers’ anger about infant sleep. Additional support to reduce doubts about managing infant sleep and further study of differences in mothers’ and fathers’ cognitive responses to children’s sleep problems would improve the effectiveness of sleep interventions.

## Abbreviations

CESD: Center for Epidemiologic Studies Depression Measure
MAF: Multidimensional Assessment of Fatigue Scale
MCISQ: Maternal (parental) Cognitions about Infant Sleep Questionnaire
PSQI: Pittsburgh Sleep Quality Index
