# Erratum to: Relationships between parental sleep quality, fatigue, cognitions about infant sleep, and parental depression pre and post-intervention for infant behavioral sleep problems

**DOI:** 10.1186/s12884-017-1386-5

**Published:** 2017-06-19

**Authors:** Wendy A. Hall, Melissa Moynihan, Radhika Bhagat, Joanne Wooldridge

**Affiliations:** 1 University of British Columbia School of Nursing, T. 201, 2211 Wesbrook Mall, Vancouver, BC V6T 2B5 Canada; 2 School of Nursing, University of British Columbia, Vancouver, BC Canada; 3 South Community Health Centre,Vancouver Coastal Health, Vancouver, BC Canada; 4 Maternal Child Program, Vancouver Coastal Health, Vancouver, BC Canada

## Erratum

Upon publication of the original article [1], it was noticed that in the discussion section, the sentence “Setting sleep limits contributed to variance in depression scores for mothers and fathers only at follow-up and for mothers, the relationship was negative (more difficulty setting sleep limits associated with lower depression scores)” was incorrectly given as “Setting sleep limits contributed to variance in depression scores for mothers and fathers only at follow-up and for mothers, the relationship was negative (less difficulty setting sleep limits associated with lower depression scores)”. This has now been acknowledged and corrected in this erratum.
